# Does the Protective Effect of Zinc on Telomere Length Depend on the Presence of Hypertension or Type 2 Diabetes? Results from the Iwaki Health Promotion Project, Japan

**DOI:** 10.3390/nu15204373

**Published:** 2023-10-16

**Authors:** Mahiro Sato, Kyi Mar Wai, Ken Itoh, Yichi Yang, Yuka Uchikawa, Yukihiko Ito, Shigeyuki Nakaji, Kazushige Ihara

**Affiliations:** 1 School of Medicine, Hirosaki University, Hirosaki 036-8562, Japan; h20m1052@hirosaki-u.ac.jp; 2 Department of Social Medicine, Graduate School of Medicine, Hirosaki University, Hirosaki 036-8562, Japanitoyukihiko@gmail.com (Y.I.);; 3 Department of Stress Response Science, Center for Advanced Medical Research, Graduate School of Medicine, Hirosaki University, Hirosaki 036-8562, Japan; 4 Research and Development Division, MiRTeL Company Limited, Hiroshima 734-0001, Japan; yuka_uchikawa@mirtel.co.jp; 5 Innovation Center for Health Promotion, Graduate School of Medicine, Hirosaki University, Hirosaki 036-8562, Japan

**Keywords:** telomere, zinc, hypertension, diabetes, Japan

## Abstract

Telomeres, repeated TTAGGG sequences at chromosomal ends, shorten with age and indicate cellular lifespan. Zinc can protect against telomere damage through its anti-oxidative effect. Meanwhile, telomere shortening was correlated with metabolic diseases of hypertension and type 2 diabetes. The objective of this study was to investigate whether the association between zinc and telomere length differs by the presence or absence of hypertension/type 2 diabetes. This is a cross-sectional study with 1064 participants of the Iwaki area, Japan. Multiple linear regression models were performed to test the hypothesis. A higher serum zinc concentration was significantly associated with a longer G-tail length (β = 48.11, 95% confidence intervals [CI]: 25.69, 70.54, *p* < 0.001). By multivariate linear regression analysis, there was a significant positive association between zinc and G-tail length in both hypertensive (β = 46.84, 95%CI: 9.69, 84.0, *p* = 0.014) and non-hypertensive groups (β = 49.47, 95%CI: 20.75, 78.18, *p* = 0.001), while the association was significant only in the non-diabetes group (β = 50.82, 95%CI: 27.54, 74.11, *p* < 0.001). In conclusion, higher zinc concentration was significantly associated with longer G-tail length. The protective effect of zinc on G-tail did not differ by hypertension status; however, it disappeared in individuals with type 2 diabetes.

## 1. Introduction

Telomeres are essential nucleoprotein structures of the repeated sequences, 5′-TTAGGG-3′, at the ends of all eukaryotic chromosomes [1,2]. G-tails are 75–300 bases of a G-rich single-stranded 3′ overhang following the telomeres [3]. Telomeres protect against genomic degradation, unnecessary recombination, repair, and interchromosomal fusion [4,5]. Meanwhile, telomeric G-tails play a pivotal role in maintaining the intramolecular loop structure of the telomere, a structure that is also known as the “t loop” [6]. The G-tail is also important to control telomerase activity, and protect the chromosome ends from fusion and degradation [3]. It also involves telomere–telomere interactions during meiosis, attaching the chromosomes to specific nuclear sites, thereby influencing telomere length maintenance [3]. In most proliferating cells, telomere length is dynamic, and the lengths of telomeres decrease gradually by 20 to 200 base pairs with each cell division in human somatic cells because of the end-replication problem [7]. In addition to the end-replication problem, external factors can shorten telomere length. For example, telomere attrition is associated with oxidative stress, inflammation, and other cellular DNA damage [7,8]. On the other hand, the G-tail could fluctuate upon exposure to various stresses [9].

Telomere length and G-tail length shorten in various metabolic diseases [9,10,11,12]. Chronic inflammation, accompanying increased cell proliferation, results in loss of telomere repeats [13]. It also has been shown that the longer the duration of diseases, the shorter the telomere length [14]. Several papers have reported that hypertensive individuals are associated with a shorter telomere length [13,15,16]. Having diabetes is also associated with a shorter telomere length, regardless of type 1 and type 2 diabetes [17,18,19]. Similarly, a previous study in hemodialysis patients reported that a shorter G-tail length is also associated with future cardiovascular events [20]. It has also been shown that a shorter G-tail length is associated with an increased risk of esophageal cancer recurrence [21]. Meanwhile, healthy lifestyles and antioxidants may positively influence telomere maintenance.

Zinc, on the other hand, plays an important role in the human body, being required for the function of about 300 enzymes [22]. Zinc is also necessary for cellular division and repair through playing an important role in DNA replication and synthesis [22,23]. It is a cofactor for numerous enzymes involved in various metabolic pathways, including cellular division, repair and growth [22,23]. DNA polymerase, RNA polymerase, and reverse transcriptase are well-known zinc-dependent enzymes, and providing zinc-rich cellular culture medium enhances telomerase activity [24]. Moreover, zinc is an essential trace element that suppresses reactive oxygen species (ROS) production, protecting against oxidative stress damage [25]. Zinc-associated proteins, such as metallothionein and copper/zinc superoxide dismutase (SOD), act as potent antioxidants in removing hydroxyl radicals or superoxide anions [23,26]. Thus, it could be hypothesized that zinc may prevent telomere attrition via telomerase and/or oxidative stress pathways.

Considering telomere length as a critical biomarker of biological aging, knowledge of the protective effect of zinc on telomeres attrition is relevant. In fact, telomeres are susceptible to damage from oxidative stress due to their abundance of guanine [5,7]. Crucially, zinc acts as a micronutrient with antioxidant properties, being a safeguard for telomeres against oxidative stress [22]. Although a couple of studies have been conducted regarding the association between zinc and telomere length, the results were inconsistent [27,28,29,30,31,32,33]. In addition, no study has examined whether the association between zinc and telomere length varies by having hypertension or type 2 diabetes mellitus. Therefore, the aim of this study was to investigate the effect of zinc on telomere length and G-tail length in the general Japanese population. This study also determined whether the association between zinc and telomere length/G-tail length differs by having the chronic diseases of hypertension and type 2 diabetes mellitus.

## 2. Materials and Methods

### 2.1. Study Design and Population

This study was a cross-sectional study using data from the Iwaki Health Promotion Project (IHPP). The IHPP is an annual health survey that has been conducted since 2005 in the Iwaki district of Hirosaki City, Aomori Prefecture, aiming to improve the life expectancy ranking in Aomori Prefecture [34].

In 2019, a total of 1073 participants (>19 years) were enrolled in the IHPP, and this study utilized the data of the 2019 IHPP. Eight participants who were absent for health check-ups were excluded. One participant with missing data on blood trace elements and telomere length was also excluded. A total of 1064 subjects were included in the final analysis.

The study was approved by the Research Ethics Committee of the Graduate School of Medicine, Hirosaki University (Approval No: 2019-009). Written informed consent was obtained from all the study participants.

### 2.2. Questionnaire, Biochemical and Anthropometric Measurements

Self-reported questionnaires were sent to the participants before the health check-ups and collected in advance. The research team checked and confirmed any insufficiency or mistakes in the responses in the face-to-face interviews on the day of the health check-ups. Information including age, sex, educational history, and lifestyle (smoking, alcohol, exercise) was collected using a questionnaire.

Blood pressure was measured using an automatic sphygmomanometer (Omron Healthcare Co., Ltd., Kyoto, Japan) on the arm with each subject in a sitting position. After the participant had rested for a few minutes, both systolic and diastolic blood pressures were measured twice, and an average of two measurements was used for the analysis.

Venous blood samples were collected under fasting conditions; the samples were stored at −20 °C or −80 °C until laboratory analysis. Fasting blood sugar, HbA1c, low-density cholesterol (LDL), high-density cholesterol (HDL), and triglycerides (TG) were measured by an enzymatic method at an accredited laboratory (LSI Medience CO., Ltd. Tokyo, Japan). Detailed measurement procedures can be found online (https://data.medience.co.jp/guide/, accessed on 21 September 2023). Height and weight were measured, and body mass index (BMI) was calculated as weight (kg) divided by height (m) squared.

### 2.3. Serum Zinc Measurement

Serum separation was performed within one to two hours after blood collection. Samples were kept in −20 °C cold storage until measurement. Serum zinc concentration was measured using a Fast Sequential Atomic Absorption Spectrophotometer (240FS AA, Agilent Technologies, Tokyo, Japan) at the laboratory (LSI Medience CO., Ltd. Tokyo, Japan) and expressed as μg/dL. Method validity was evaluated by measuring the precision of control samples before and after to confirm that they were within the default range.

### 2.4. Definitions of Hypertension and Type 2 Diabetes Mellitus

In this study, hypertension was defined as systolic blood pressure ≥ 140 mmHg, diastolic blood pressure ≥ 90 mmHg, or the use of antihypertension medication by the definition of the Japanese Society of Hypertension. Diabetes mellitus was defined as a fasting blood sugar level ≥ 126 mg/dL and HbA1c ≥ 6.5% or the use of diabetes medication, according to the Japanese Diabetes Society. Dyslipidemia was defined as LDL cholesterol level ≥ 140 mg/dL, HDL cholesterol level < 40 mg/dL, triglyceride level ≥ 150 mg/dL, or the use of antihyperlipidemic medication in accordance with the Japan Atherosclerosis Society.

### 2.5. Telomere Length Measurement

Total telomere length and G-tail length were measured by using Hybridization Protection Assay (HPA). Total telomere length is the total length, including the G-tail, the single chain at the end of the telomere. Total telomere length was assessed by stripping the double strand at high temperature and reacting (hydrolyzing) the probe, while G-tail was measured without stripping the double strand. The detailed methods of measurement are described in the previously published literature [11]. Coefficients of variables were less than 5% for both total telomere length and G-tail measurements.

### 2.6. Statistical Analysis

All statistical analyses were performed using Stata Version 16 (StataCrop LLC, College Station, TX, USA). Descriptive analyses were expressed as means, standard deviations, median, interquartile range, or percentage. Pearson’s correlation test and Spearman’s rank correlation coefficient were performed to examine the correlation of telomere length and G-tail length with serum zinc and other variables according to the data distribution. Multivariate linear regression models were performed to examine whether there were differences in the associations of serum zinc and telomere length/G-tail length by hypertension or diabetes groups. Model 1 was unadjusted, and Model 2 was adjusted for age, gender, education, smoking history, drinking history, exercise habits, BMI, hypertension, diabetes, and dyslipidemia as confounding factors. As a sensitivity analysis, separate models were constructed and analyzed with different definitions of hypertension and diabetes by excluding medically controlled individuals for a better understanding of the associations at the biochemical level. This could help to determine whether the study results were robust regardless of subjective data on medication history or objective data on blood pressure, serum sugar, and HbA1c. In fact, blood pressure, serum sugar, and HbA1c data were based on the measurements of blood samples on the health check-up day, which may underrepresent the chronic effect on telomere. Thus, our principal analysis included the data under the definitions of hypertension and diabetes, inclusive of medication history, to cover the chronic effect. In all statistics, a *p*-value of <0.05 was considered significant.

## 3. Results

The characteristics of the participants are presented in Table 1. The mean age was 52.7 years (standard deviation [SD] = 15.3 years). The average telomere length and G-tail length were 356,478.1 RLU/mg DNA (SD = 50,678.4 RLU/mg DNA) and 31,664.2 RLU/mg DNA (SD = 4744.9 RLU/mg DNA), respectively. The mean serum zinc concentration was 84.8 μg/dL (SD = 12.9 μg/dL). Of all participants, 35.5% had hypertension, and 6.7% had type 2 diabetes mellitus.

Table 2 shows the correlation between study characteristics and telomere length/G-tail length. Telomere length and G-tail length are positively correlated (r = 0.38, *p* < 0.001). Both telomere length (r = −0.40, *p* < 0.001) and G-tail length (r = −0.24, *p* < 0.001) shorten with increasing age. There is a significant positive correlation between serum zinc and G-tail length (r = 0.151, *p* < 0.001). Increased blood pressure or serum sugar was negatively correlated with telomere length or G-tail length.

Table 3 presents the association between serum zinc and telomere length/G-tail length by multivariable linear regression. Serum zinc had a significant positive association with G-tail length (adjusted β = 48.11, 95%CI 25.69~70.54, *p* < 0.001), while it showed a positive nonsignificant association with TL (adjusted β = 18.4, 95%CI −208.57~245.37, *p* = 0.874).

**Figure 1:**
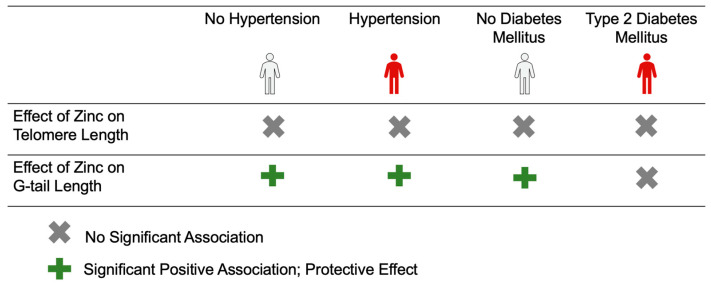
A Summarized Illustration of the Effect of Zinc on Telomere/G-tail Length as Stratified by Hypertension or Type 2 Diabetes Mellitus (N = 1064).

Table 4 and Table 5 show the associations between serum zinc and telomere length/G-tail length stratified by hypertension. When the study participants were stratified into hypertensive and non-hypertensive groups, there was a significant positive association between serum zinc and G-tail length in both hypertensive (adjusted β = 46.84, 95%CI: 9.69, 84.00, *p* = 0.014) and non-hypertensive groups (adjusted β = 49.47, 95%CI: 20.75, 78.18, *p* = 0.001). However, as shown in Table 6 and Table 7, the association was significant only in the non-diabetes mellitus group (adjusted β = 50.82, 95%CI: 27.54, 72.67, *p* < 0.001), not in the diabetic group (adjusted β = 33.63, 95%CI: −63.62, 130.87, *p* = 0.491) when stratified by diabetes. There was no significant association between serum zinc concentration and telomere length in both groups of hypertension or diabetes. Figure 1 illustrated the summarized results for the effect of zinc on telomere/G-tail length by hypertension or type 2 diabetes mellitus.

The sensitivity analysis shows similar results as the main analysis. The protective effect of zinc on the G-tail did not differ by hypertension, but it was concealed in the type 2 diabetes individuals (Supplementary Table S1).

## 4. Discussion

The current study identified a significant positive association between serum zinc concentration and G-tail length in the general Japanese population. This positive association was consistent in both hypertension and non-hypertension subjects. However, when stratified by diabetes status, zinc showed a significant positive association with G-tail length only in non-diabetic subjects.

To the best of our knowledge, the association between zinc and G-tail length is a novel finding. In this study, a higher serum zinc concentration was significantly associated with a longer G-tail length. Although the previous studies examined the association between zinc and telomere length, none specifically explained the link between zinc and G-tail length, a terminal overhang of the telomere. For example, a randomized control study conducted on the elderly (65–85 years) in South Australia reported that telomere length was increased with zinc supplementation [27]. Similarly, a previous cross-sectional study revealed that dietary zinc intake was correlated with longer telomere length among U.S. adults [29]. Some studies also showed no significant association between zinc and telomere length [30,32,33]. The inconsistencies could be partly explained by the fact that the prior studies estimated the zinc level from the dietary intake [29,30]. In contrast, the current study measured the serum zinc concentration of the general population. No population studies have yet explained the association between zinc and G-tail length, although G-tail is a nimbler sensor for the external factors than the entire telomere [9]. Our results provide the original finding that zinc is positively associated with G-tail length in the general population-based setting.

The following mechanisms may explain why higher serum zinc is associated with longer G-tail length. Importantly, zinc is an antioxidant micronutrient and has a protective role in oxidative stress damage [25]. In particular, zinc is a structural component of the enzyme of SOD, which is a potent factor in reducing ROS production by converting harmful superoxide radicals to less harmful ones [35]. Moreover, free zinc ions induce a signaling pathway of the expression of metallothionein, a cysteine-rich metal ions-binding protein [36]. Increased expression of metallothionein possibly allows increased zinc-metallothionein formation associated with antioxidant activity via the oxidation of heavy metal-bound cysteines as a scavenging system for free radicals [35,36,37]. Telomeres are sensitive to oxidative stress damage due to their high guanine content [7]. Oxidative stress can directly attack the telomeric DNA sequence, accelerating telomere shortening and reducing their ability to protect the chromosomal ends [4,5,7]. Furthermore, oxidative stress interconnects with telomerase in the context of telomere maintenance by direct inhibition of the enzyme or through modifications of the protein involved in the telomerase complex [7,13]. ROS influences the expression and regulation of telomeres at the transcriptional and translational level, thereby affecting the expression of genes related to telomerase activity, hindering its ability to elongate telomeres [2,7]. Thus, zinc protects telomeres from oxidative stress, possibly resulting in longer telomere G-tail length at higher serum zinc concentrations. Furthermore, zinc-dependent telomerase activity involves telomere length maintenance [38]. The previous study demonstrated that zinc-rich culture medium showed increased telomerase activity [38], zinc being a component of reverse transcriptase enzyme. Another explanation could be the direct effect of zinc on the genomic integrity of telomeric DNA. Zinc deficiency has been associated with DNA repair via p53 tumor suppressor proteins regulation [23]. Zinc also regulates the activity of poly(ADP-ribose) polymerase, thereby being involved in the role of DNA break recognition [23,24]. Thus, our findings of the positive association between zinc and G-tail length are reasonable, as supported by the pathways mentioned above.

In this study, zinc is positively, although not significantly, associated with telomere length. In other words, we found a significant positive link between zinc and G-tail length but not with telomere length. The result is similar to previous studies that have examined the associations between cardiovascular events, breast cancer, and telomere, where only the G-tail length showed a significant association [20,39]. Differences in association with zinc between telomere length and G-tail length are possibly due to the differences in their respective sensitivities. The G-tail is more sensitive to pathophysiological stresses than the whole telomere [9]. Furthermore, the G-tail constitutes 70–300 base pairs, which is much shorter than telomere length [40]. The variation in G-tail length by serum zinc may have little change in total telomere length. Therefore, our result suggests that the G-tail is more sensitive to the effect of zinc than telomere length.

In this study, both hypertensive and non-hypertensive groups showed significant positive associations between serum zinc and G-tail length. However, the effect size is smaller in the hypertensive group than in the non-hypertensive group, indicating that the protective effect of zinc on G-tail length was weakened in hypertensive individuals. In the previous meta-analysis of the association between hypertension and telomere length, telomere length was significantly shorter in the hypertension group than in the control group [15]. It was also reported that cardiovascular risk factors, including hypertension, are associated with short leukocyte telomere length [13]. Considering hypertension itself may have a direct negative effect on the telomere, it is plausible that the protective effect of zinc on G-tail length is diminished in the hypertensive group. On the other hand, our results show that zinc has a protective effect on G-tail length regardless of whether individuals have hypertension.

This study also revealed that the association between serum zinc and G-tail length was not significant in the type 2 diabetes group, although there was a significant association in the non-diabetic group. This could be explained by the increased oxidative stress status in type 2 diabetes mellitus individuals. In fact, hyperglycemia induces systemic oxidative stress, which in turn induces telomere attrition [17]. Telomere attrition in adipocytes induces insulin resistance, leading to further hyperglycemia [17]. In other words, a vicious cycle of telomere attrition due to hyperglycemia is built in diabetic individuals. Moreover, zinc plays a critical role in insulin synthesis, secretion, and storage, and lower zinc status was known to be associated with a higher diabetes prevalence [41]. In diabetes individuals, intestinal zinc malabsorption or hyperglycemia provokes an increase in urinary loss of zinc and a decrease in total body zinc, which leads to impaired zinc homeostasis [41,42]. The present results suggest that zinc demonstrates a protective effect on G-tail length; however, in diabetic individuals, either the increased demand for zinc is required, or the oxidative stress status is elevated beyond the range that zinc can compensate for.

Some limitations should be considered in this study. The present study was limited by its cross-sectional nature, which prevents inferring causation. Therefore, longitudinal studies are needed to confirm the role of zinc on telomere length or G-tail length. Our results may not necessarily be generalizable to other populations because the study subjects reside in a limited area, i.e., the Iwaki district. Moreover, the HPA method was used for telomere assessment in this study, although terminal restriction fragment analysis is the gold standard [43]. The study is also limited by including the dietary zinc intake or dietary history, which could have some effect on the variables of interest.

## 5. Conclusions

In conclusion, this study identified that higher serum zinc concentration is significantly associated with a longer G-tail length in a general Japanese population. The association did not differ by hypertension, suggesting that zinc may protect G-tail length erosion. However, the protective effect of zinc on G-tail length is not evident in diabetic individuals. Therefore, it is recommended that diabetic individuals ensure proper zinc homeostasis in their bodies while also focusing on diabetes control to reap the protective effect of zinc on telomeres.

## Figures and Tables

**Table 1:** Descriptive Results of the Study Participants (N = 1064).

**Variables**	**Mean**	**Standard Deviation**
Age (years)	52.7	15.3
Body Mass Index: BMI (kg/m^2^)	23.0	3.6
Telomere length (RLU/mg DNA)	356,478.1	50,678.4
G-tail length (RLU/mg DNA)	31,664.2	4744.9
Systolic blood pressure (mmHg)	120.8	16.9
Diastolic blood pressure (mmHg)	76.9	11.4
HbA1c (%) ‡	5.6	0.4
Serum sugar (mg/dL) ‡	93.0	133.0
Serum zinc (μg/dL)	84.8	12.9
	**N**	**(%)**
Sex		
Male	435	40.9
Female	629	59.1
Education		
Junior high school	112	10.5
High school	577	54.2
Junior college	248	23.3
University/college	117	11.0
Others	6	0.6
Smoking status		
No	673	63.3
Current	182	17.1
Past	199	18.7
Drinking status		
No	503	47.3
Current	504	47.4
Past	43	4.0
Physical exercise (Other than winter)	234	22.0
Physical exercise (winter)	230	21.6
Hypertension	378	35.5
Type 2 diabetes mellitus	71	6.7
Dyslipidemia	399	37.5
Currently taking anti-hypertensive drugs	247	23.2
Currently taking anti-diabetic drugs	49	4.6
Currently taking anti-dyslipidemia drugs	107	9.8

‡ Median and interquartile range are displayed.

**Table 2:** Correlations between Study Characteristics and Telomere Length (N = 1064).

	**Telomere Length**		**G-Tail Length**	
**Variables**	**Pearson’s γ**	***p*-Value**	**Pearson’s γ**	***p*-Value**
Age (years)	−0.4	<0.001	−0.24	<0.001
Systolic blood pressure (mmHg)	−0.157	<0.001	−0.106	<0.001
Diastolic blood pressure (mmHg)	−0.072	0.019	−0.036	0.243
Body mass index (kg/m^2^)	−0.113	<0.001	−0.006	0.854
Serum zinc (μg/dL)	0.033	0.287	0.151	<0.001
G-tail length (RLU/mg DNA)	0.38	<0.001		
	**Spearman’s Rho**	***p*-Value**	**Spearman’s Rho**	***p*-Value**
HbA1c (%)	−0.257	<0.001	−0.125	<0.001
Serum sugar (mg/dL)	−0.209	<0.001	−0.084	0.006

**Table 3:** Associations between Serum Zinc and Telomere Length and G-tail by Linear Regression (N = 1064).

Variables	Telomere Length		G-tail Length	
	Model 1	Model 2	Model 1	Model 2
	β (95%CI)	β (95%CI)	β (95%CI)	β (95%CI)
Serum zinc (μg/dL)	128.8 (−108.47, 366.06)	18.4 (−208.57, 245.37)	55.69 (33.72, 77.66) ***	48.11 (25.69, 70.54) ***
Age (years)		−1321.38 (−1546.27, −1096.3) ***		−76.22 (−98.45, −53.99) ***
Sex (ref: Male)				
Female		3626.97 (−3027.58, 10,281.51)		17.13 (−640.39, 674.66)
Education (ref: below junior college)				
Junior college and above		−1882.05 (−8071.28, 4307.18)		−41.64 (−653.19, 569.90)
Smoking (ref: Non-smokers)				
Current smokers		−9761.76 (−18,281.73, −1241.79) *		−59.40 (−901.24, 782.45)
Past smokers		−3742.33 (−11,615.11, 4130.45)		−472.04 (−1249.94, 305.86)
Alcohol drinking (ref: Non-drinkers)				
Current drinkers		−2264.24 (−8646.38, 4117.89)		−88.59 (−719.20, 542.02)
Past drinkers		698.24 (−13,979.32, 15,375.81)		744.92 (−705.34, 2195.19)
Physical exercise (ref: No)		−183.31 (−7711.99, 7345.38)		−674.80 (−1418.69, 69.10)
Body mass index (kg/m^2^) (ref: <18.5 kg/m^2^)				
18.5 or 18.5–25 kg/m^2^		−2542.98 (−13,427.81, 8341.86)		646.28 (−429.23, 1721.80)
≥25 kg/m^2^		−5138.97 (−17,226.54, 6948.60)		562.82 (−631.53, 1757.17)
Hypertension (ref: No)		2597.17 (−4280.28, 9474.63)		−25.67 (−705.22, 653.88)
Type 2 diabetes mellitus (ref: No)		−18,999.33 (−30,833.92, −7164.73) **		260.66 (−908.70, 1430.01)
Dyslipidemia (ref: No)		−2419.61 (−8771.77, −3932.55)		374.69 (−252.96, 1002.34)

CI: Confidence Intervals; * *p*-value < 0.05; ** *p*-value < 0.01; *** *p*-value < 0.001; Model 1: Unadjusted; Model 2: Adjusted for age, sex, education, smoking status, drinking status, exercise, body mass index, hypertension, diabetes mellitus, dyslipidemia.

**Table 4:** Associations between Serum Zinc and Telomere Length Stratified by Hypertension (N = 1064).

Variables	No Hypertension N = 686		Hypertension N = 378	
	Model 1	Model 2	Model 1	Model 2
	β (95%CI)	β (95%CI)	β (95%CI)	β (95%CI)
Serum zinc (μg/dL)	78.96 (−218.05, 375.97)	50.89 (−237.35, 339.13)	148.10 (−228.89, 525.08)	−46.11 (−428.44, 336.23)
Age (years)		−1274.38 (−1553.51, −995.28) **		−1470.51 (−1893.65, −1047.36) **
Sex (ref: Male)				
Female		2814.95 (−5621.74, 11251.65)		6106.05 (−5264.46, 17476.56)
Education (ref: below junior college)				
Junior college and above		−173.93 (−7768.76, 7420.91)		−6866.10 (−18,102.29, 4370.10)
Smoking (ref: Non-smokers)				
Current smokers		−10,668.75 (−27748.21, −589.3) *		−8811.48 (−25,681.46, 8058.50)
Past smokers		−5571.46 (−15,543.24, 4400.32)		684.60 (−12,384.35, 13,753.56)
Alcohol drinking (ref: Non-drinkers)				
Current drinkers		−2597.51 (−10,406.51, 5211.49)		−3044.71 (−148,04.48, 8715.06)
Past drinkers		−4565.16 (−22,919.42, 13,789.10)		12,006.70 (−13,108.77, 37,122.16)
Physical exercise (ref: No)		2258.92 (−7368.96, 11,886.79)		−5096.39 (−17,523.87, 7331.09)
Body mass index (kg/m^2^) (ref: <18.5 kg/m^2^)				
18.5 or 18.5–25 kg/m^2^		−5041.06 (−17,579.15, 7497.02)		3200.69 (−20,175.63, 26,577.02)
≥25 kg/m^2^		−4350.55 (−19,115.25, 10,454.14)		−4023.92 (−28,019.93, 19,972.10)
Type 2 diabetes mellitus (ref: No)		−24,322.36 (−45,408.30, −3236.42) *		−16,994.83 (−31,321.67, −2667.99) *
Dyslipidemia (ref: No)		−2248.33 (−10,938.59, 6441.94)		−2814.97 (−12,611.19, 6981.25)

CI: Confidence Intervals; * *p*-value < 0.05; ** *p*-value < 0.001. Model 1: Unadjusted; Model 2: Adjusted for age, sex, education, smoking status, drinking status, exercise, body mass index, diabetes mellitus, dyslipidemia.

**Table 5:** Associations between Serum Zinc and G-tail Length Stratified by Hypertension (N = 1064).

Variables	No Hypertension N = 686		Hypertension N = 378	
	Model 1	Model 2	Model 1	Model 2
	β (95%CI)	β (95%CI)	β (95%CI)	β (95%CI)
Serum zinc (μg/dL)	52.90 (24.94, 80.86) ***	49.47 (20.75, 78.18) **	57.04 (22.01, 92.08) ***	46.84 (9.69, 84.00) *
Age (years)		−72.99 (−100.80, −45.18) ***		−88.25 (−129.37, −47.12) ***
Sex (ref: Male)				
Female		257.68 (−582.90, 1098.26)		−494.67 (−1599.65, 610.32)
Education (ref: below junior college)				
Junior college and above		−126.64 (−883.34, 630.06)		−38.66 (−1130.60, 1053.27)
Smoking (ref: Non-smokers)				
Current smokers		5.79 (−998.46, 1010.05)		−420.91 (−2060.33, 1218.51)
Past smokers		−655.54 (−1649.06, 337.99)		−207.28 (−1477.32, 1062.76)
Alcohol drinking (ref: Non-drinkers)				
Current drinkers		79.23 (−698.81, 857.27)		−581.51 (−1724.32, 561.31)
Past drinkers		772.30 (−1056.41, 2601.00)		749.43 (−1691.29, 3190.15)
Physical exercise (ref: No)		−837.25 (−1796.51, 122.01)		−406.62 (−1614.32, 801.09)
Body mass index (kg/m^2^) (ref: <18.5 kg/m^2^)				
18.5 or 18.5–25 kg/m^2^		972.22 (−277.00, 2221.43)		−549.98 (−2821.69, 1721.73)
≥25 kg/m^2^		1270.78 (−204.27, 2745.82)		−1096.55 (−3428.48, 1235.38)
Type 2 diabetes mellitus (ref: No)		−225.87 (−2326.74, 1875.00)		480.81 (−911.47, 1873.09)
Dyslipidemia (ref: No)		294.84 (−571.00, 1160.68)		315.29 (−636.70, 1267.29)

CI: Confidence Intervals; * *p*-value < 0.05; ** *p*-value < 0.01; *** *p*-value < 0.001. Model 1: Unadjusted; Model 2: Adjusted for age, sex, education, smoking status, drinking status, exercise, body mass index, diabetes mellitus, dyslipidemia.

**Table 6:** Associations between Serum Zinc and Telomere Length Stratified by Type 2 Diabetes Mellitus (N = 1064).

Variables	No Diabetes Mellitus N = 993		Type 2 Diabetes Mellitus N = 71	
	Model 1	Model 2	Model 1	Model 2
	β (95%CI)	β (95%CI)	β (95%CI)	β (95%CI)
Serum zinc (μg/dL)	132.76 (−108.81, 374.34)	40.29 (−194.71, 275.28)	−389.19 (−1373.16, 594.79)	−355.88 (−1399.54, 687.79)
Age (years)		−1291.28 (−1524.56, −1058.00) *		−1792.54 (−2867.96, −717.11)
Sex (ref: Male)				
Female		3287.20 (−3661.98, 10,236.38)		11,391.46 (−17,304.77, 40,087.68)
Education (ref: below junior college)				
Junior college and above		−1297.31 (−7691.85, 5097.23)		−11,415.26 (−38,286.81, 15,456.30)
Smoking (ref: Non-smokers)				
Current smokers		−8639.30 (−17462.37, 183.78)		−21,291.34 (−57,760.54, 15,177.87)
Past smokers		−4541.19 (−12,680.77, 3598.39)		18,358.79 (−17,623.85, 54,341.43)
Alcohol drinking (ref: Non-drinkers)				
Current drinkers		−1980.46 (−8542.66, 4581.74)		−14,222.07 (−43656.04, 15211.89)
Past drinkers		−1757.56 (−17,387.16, 13,872.03)		22,346.24 (−29005.18, 73697.66)
Physical exercise (ref: No)		−2030.63 (−9841.42, 5780.16)		23,971.98 (−7722.96, 55666.93)
Body mass index (kg/m^2^) (ref: <18.5 kg/m^2^)				
18.5 or 18.5–25 kg/m^2^		−2987.63 (−13,991.04, 8015.84)		19,289.75 (−80,950.12, 119,529.60)
≥25 kg/m^2^		−4208.25 (−16,576.26, 8159.76)		−6057.40 (−107,536.20, 95,421.45)
Hypertension (ref: No)		2183.22 (−5036.14, 9402.58)		8282.28 (−17,365.05, 33,929.60)
Dyslipidemia (ref: No)		−4333.54 (−10,966.07, 2299.00)		23,947.59 (678.77, 47,216.41)

CI: Confidence Intervals; * *p*-value < 0.001. Model 1: Unadjusted; Model 2: Adjusted for age, sex, education, smoking status, drinking status, exercise, body mass index, hypertension, dyslipidemia.

**Table 7:** Associations between Serum Zinc and G-tail Length Stratified by Type 2 Diabetes Mellitus (N = 1064).

Variables	No Diabetes Mellitus N = 993		Type 2 Diabetes Mellitus N = 71	
	Model 1	Model 2	Model 1	Model 2
	β (95%CI)	β (95%CI)	β (95%CI)	β (95%CI)
Serum zinc (μg/dL)	57.93 (35.26, 80.59) *	50.82 (27.54, 74.11) **	11.85 (−81.43, 105.13)	33.63 (−63.62, 130.87)
Age (years)		−67.89 (−91.01, −44.77) **		−197.49 (−297.69, −97.28) **
Sex (ref: Male)				
Female		−26.28 (−714.94, 662.38)		−417.87 (−3091.69, 2255.96)
Educational level (ref: below junior college)				
Junior college and above		60.93 (−572.76, 694.63)		−2141.87 (−4645.67, 361.94)
Smoking (ref: Non-smokers)				
Current smokers		−127.21 (−1001.57, 747.15)		312.27 (−3085.82, 3710.35)
Past smokers		−677.16 (−1483.79, 129.47)		2627.06 (−725.69, 5979.81)
Alcohol drinking (ref: Non-drinkers)				
Current drinkers		−6.82 (−657.13, 643.49)		−2382.77 (−5125.34, 359.79)
Past drinkers		584.48 (−964.40, 2133.36)		2149.24 (−2635.53, 6934.01)
Physical exercise (ref: No)		−768.01 (1542.05, 6.04)		1227.39 (−1725.85, 4180.62)
Body mass index (kg/m^2^) (ref: <18.5 kg/m^2^)				
18.5 or 18.5–25 kg/m^2^		589.67 (−500.76, 1680.10)		4467.88 (−4872.16, 13807.93)
≥25 kg/m^2^		488.61 (−737.05, 1714.27)		3138.96 (−6316.53, 12594.45)
Hypertension (ref: No)		−153.38 (−868.81, 562.06)		1298.06 (−1091.68, 3687.80)
Dyslipidemia (ref: No)		284.32 (−372.96, 941.70)		1267.91 (−900.21, 3436.03)

CI: Confidence Intervals; * *p*-value < 0.01; ** *p*-value < 0.001. Model 1: Unadjusted; Model 2: Adjusted for age, sex, education, smoking status, drinking status, exercise, body mass index, hypertension, dyslipidemia

## Data Availability

The data presented in this study are available on request from the corresponding author (K. Ihara: ihara@hirosaki-u.ac.jp) upon reasonable request.

## Supplementary Materials

The following supporting information can be downloaded at: https://www.mdpi.com/article/10.3390/nu15204373/s1, Table S1: Association between Serum Zinc and Telomere Length/G-tail Length Stratified by Hypertension and Diabetes Mellitus (N = 1064).
