# Supplementary material for: Does the Protective Effect of Zinc on Telomere Length Depend on the Presence of Hypertension or Type 2 Diabetes? Results from the Iwaki Health Promotion Project, Japan
Source: Nutrients. 2023 Oct 16;15(20):4373. doi: 10.3390/nu15204373 (PMC10609662; doi:10.3390/nu15204373)
Supplement: Supplementary file 1 [file nutrients-15-04373-s001.zip › nutrients-2652732-supplementary.pdf]

Supplementary Table S1: Association between Serum Zinc and Telomere Length/G-tail Length Stratified by Hypertension and Diabetes Mellitus (N=1064)

**Variable**	**No Hypertension (N=862)**		**Hypertension (N=202)**	
	**Model 1**	**Model 2**	**Model 1**	**Model 2**
	**β (95%CI)**	**β (95%CI)**	**β (95%CI)**	**β (95%CI)**
Telomere Length as an outcome				
Serum zinc (μg/dL)	164.45(-103.49, 432.39)	61.59(-196.58, 319.77)	65.03(-437.90, 567.96)	-231.05(-756.44, 294.34)
G-tail Length as an outcome				
Serum zinc (μg/dL)	50.99(26.11, 75.87) ***	46.17(20.64, 71.62) ***	77.55(30.89, 121.21) **	65.01(14.40, 115.79) *

**Variable**	**No Diabetes Mellitus (N=1025)**		**Diabetes Mellitus (N=39)**	
	**Model 1**	**Model 2**	**Model 1**	**Model 2**
	**β (95%CI)**	**β (95%CI)**	**β (95%CI)**	**β (95%CI)**
Telomere Length as an outcome				
Serum zinc (μg/dL)	101.73(-138.91, 342.37)	15.27(-217.99, 248.52)	-25.35(-1376.11, 1325.41)	-83.45(-1802.88, 1635.98)
G-tail Length as an outcome				
Serum zinc (μg/dL)	57.39(35.12, 79.66) ***	51.98(29.08, 74.88) ***	-4.40(-152.93, 144.13)	-35.57(-184.13, 112.99)

CI: Confidence Intervals; **p*-value<0.05; ***p*-value<0.01; ****p*-value<0.001; Model 1: Unadjusted; Model 2: Adjusted for age, sex, education, smoking status, drinking status, exercise, body mass index, and dyslipidemia; Hypertension is defined as systolic blood pressure ≥ 140 mmHg and/or diastolic blood pressure ≥ 90 mmHg; Diabetes is defined as a fasting blood sugar level ≥ 126 mg/dL and HbA1c ≥ 6.5%.
